# Intensified household contact tracing, prevention and treatment support versus enhanced standard of care for contacts of tuberculosis cases in South Africa: study protocol for a household cluster-randomised trial

**DOI:** 10.1186/s12879-019-4502-5

**Published:** 2019-10-12

**Authors:** Peter MacPherson, Emily L. Webb, Ebrahim Variava, Sanjay G. Lala, Minja Milovanovic, Andrew Ratsela, Limakatso Lebina, Anthony Kinghorn, Neil A. Martinson

**Affiliations:** 1 Malawi-Liverpool-Wellcome Trust Clinical Research Programme, Queen Elizabeth Central Hospital, Blantyre, Malawi; 2 Department of Clinical Sciences, Liverpool School of Tropical Medicine, Liverpool, UK; 3 MRC Tropical Epidemiology Group, London School of Hygiene and Tropical Medicine, London, UK; 4 Department of Internal Medicine, Klerksdorp Tshepong Hospital Complex and University of the Witwatersrand, Klerksdorp, South Africa; 5 Perinatal HIV Research Unit (PHRU), SA MRC Soweto Matlosana Collaborating Centre for HIV/AIDS and TB, University of the Witwatersrand, Soweto, South Africa; 6 Department of Paediatrics, Chris Hani Baragwanath Academic Hospital, and University of the Witwatersrand, Soweto, South Africa; 7 Department of Medicine, Polokwane and Mankweng Hospitals, University of Limpopo, Polokwane, South Africa

**Keywords:** Tuberculosis, HIV, Case-finding, Prevention, Screening, Treatment, Cluster randomised trial, South Africa, Sub-Saharan Africa

## Abstract

**Background:**

Household contact tracing of index TB cases has been advocated as a key part of TB control for many years, but has not been widely implemented in many low-resource setting because of the current dearth of high quality evidence for effectiveness. Innovative strategies for earlier, more effective treatment are particularly important in contexts with hyper-endemic levels of HIV, where levels of TB infection remain extremely high.

**Methods:**

We present the design of a household cluster-randomised controlled trial of interventions aimed at improving TB-free survival and reducing childhood prevalence of *Mycobacterium tuberculosis* infection among household contacts of index TB cases diagnosed in two provinces of South Africa. Households of index TB cases will be randomly allocated in a 1:1 ratio to receive either an intensified home screening and linkage for TB and HIV intervention, or enhanced standard of care. The primary outcome will compare between groups the TB-free survival of household contacts over 15 months. All participants, or their next-of-kin, will provide written informed consent to participate.

**Discussion:**

Evidence from randomised trials is required to identify cost-effective approaches to TB case-finding that can be applied at scale in sub-Saharan Africa.

**Trial registration:**

ISRCTN16006202 (01/02/2017: retrospectively registered) and NHREC4399 (11/04/2016: prospectively registered). Protocol version: 4.0 (date: 18th January 2018).

## Background

Tuberculosis (TB) remains a public health priority, and is now the leading adult infectious cause of death worldwide [1]. In sub-Saharan Africa, TB incidence rates have skyrocketed [1, 2], driven principally by extremely high prevalence of human immunodeficiency virus (HIV) infection [2]. South Africa is the country with the largest number of people living with HIV worldwide [3], and has experienced extremely high TB incidence, prevalence and TB-related mortality [4].

The End-TB Strategy calls for bold action to eliminate tuberculosis as a public health issue by 2035 [5]. Central to the End-TB strategy is a recognition that integrated patient-centred care is required to treat all cases of tuberculosis, along with systematic screening of contacts and other high-risk groups to provide effective preventive interventions [5]. Moreover, high out-of-pocket costs of care-seeking and treatment have proved to be catastrophic for many people attempting to accesses TB care and treatment [6, 7]. Interventions that can deliver earlier, equitable, affordable and effective TB and HIV diagnosis and treatment are urgently required.

### Rationale for trial

Contact tracing of index TB cases (sometimes known as “sentinel” or “source” cases) has been advocated as a key part of TB control for many years because it facilitates early identification of symptomatic and infectious linked cases, and allows individuals with TB infection to receive preventive treatment [8–10]. Although, World Health Organization (WHO) [10] and National Guidelines in South Africa [11] recommend household contact tracing, the 2012 WHO guidelines have not been prioritized or widely implemented in South Africa (or indeed in many other high TB burden countries) because of the limited available evidence for effectiveness or cost-effectiveness [12], and the requirement to reallocate resources in many settings that are already struggling to identify, diagnose and treat people with TB symptoms [13].

The Zambia, South Africa Tuberculosis and AIDS Reduction (ZAMSTAR) community randomized trial in Zambia and South Africa [14] has shown that household contact tracing has the potential to reduce new TB infections in young children and TB prevalence in communities, whereas untargeted community TB case finding intervention also tested in ZAMSTAR had no impact on these outcomes. The ZAMSTAR household contact tracing intervention comprised of: identification of target households through adult index TB cases; symptom screening of household contacts to identify individuals for further TB investigation; testing symptomatic individuals using sputum smear microscopy. To date, the ZAMSTAR study team has not reported on process and outcome indicators of the household intervention, such as: the rates of newly diagnosed TB disease and/or HIV infection in contacts who accessed care and prevention services; the number of times that the entire household was offered TB symptom screening; the proportion of contacts who should have received TB preventive treatment and/or antiretroviral therapy (ART), and who actually received it; and the costs of implementing this strategy its cost effectiveness in relation to passive case finding. Additionally, the symptom-based, sputum smear screening strategy used in ZAMSTAR to identify and investigate TB suspects likely missed some household contacts with prevalent infectious TB [15], thereby limiting the effectiveness of the household interventions. Moreover, at the time of the trial ART - a potent means of preventing TB disease - was not universally available, with initiation of ART based on cluster of differentiation 4 (CD4) cell count thresholds.

Our prior studies in households of adult and paediatric index TB cases in two different South African settings where HIV prevalence is high, indicate that a substantial proportion of household contacts who have sputum cultures positive for *M. tuberculosis* do not report symptoms and, under the ZAMSTAR strategy, would not have been identified by symptom screening [16, 17]. Moreover, data from the Vhembe Health District in Limpopo, South Africa (a relatively low TB incidence area) showed that household contact tracing identified comparable rates of undiagnosed TB disease in contacts as in the high TB incidence area (or “hot spot” area) of Matlosana District; again, a significant proportion of contacts with newly identified culture-positive TB disease reported no symptoms [18].

Mathematical models emphasize that the failure to detect contacts with asymptomatic or subclinical TB limits the potential reduction on community TB transmission [19] and suggest that earlier identification and prompt treatment of contacts could result in improved population-level TB control [19].

Compared to sputum culture and the newer molecular TB diagnostics, sputum smear has reduced sensitivity in HIV-infected individuals because of the paucibacillary nature of early TB disease [20]. Since the final ZAMSTAR prevalence survey in 2010, routine TB diagnostics have improved markedly with the widespread scale-up of the Xpert MTB/Rif molecular TB diagnostic platform, which is more sensitive and specific than sputum smear in diagnosing TB [21]. Importantly, Xpert reduces diagnostic delay and allows for earlier TB treatment initiation [22]. TB preventive treatment guidelines for HIV-infected individuals now recommend long-term, more effective and safe regimens [23, 24], which when co-administered with antiretroviral therapy (ART) reduce mortality and TB disease [25, 26]. WHO has recommended a “test-and-treat” with immediate ART initiation for all individuals living with HIV [27]; first line ART regimens have been simplified to single daily dosing with fixed-dose combination tablets [27]. Moreover, children diagnosed with TB are increasingly being recognized as important sentinel cases for rapid identification of an infectious household TB case [17, 28] and equally importantly, for identifying undiagnosed or untreated maternal HIV infection. Finally, our data shows that HIV-seronegative household contacts in a high burden setting in the year after a household contact tracing intervention had annual TB incidence of 700/100,000 [29]. This is considerably higher than in most HIV-seronegative individuals and suggests preventive treatment against TB should be offered to HIV seronegative adults and older children, although not currently part of guidelines for preventive treatment against TB in South Africa.

We therefore hypothesise that an intensified household contact tracing and treatment support intervention that includes these diagnostic and preventative advances will improve TB-free survival among household contacts, and will decrease the prevalence of TB infection in children.

## Methods/design

### Study design

A household cluster randomised trial.

### Study setting

The study is being conducted in two South African Districts, selected based primarily on their differing TB and HIV burden and existing community research links (Botshabelo Subdistrict, Mangaung Metropolitan Municipality in the Free State, population, 2011 population: 181,712 [30]; and the Capricorn Health District in Limpopo Province, 2011 population: 1,293,000 [31]) – map: Additional file 1: Figure S1. The antenatal HIV seroprevalence and the annual TB incidence for 2012 in Mangaung Municipality and in the Capricorn Health District were 32% and 686/100,000 and 15% and 371/100,000 respectively [32, 33]. These two settings provide valuable information on the intervention when implemented in a TB “hot spot” compared to one with a lower annual TB burden.

### Identification of index TB cases

Index TB cases eligible to participate in the study will be any patient (living or recently deceased within 6 weeks of confirmed TB diagnosis) who is a permanent resident of one of the study districts with no plans to relocate over the planned duration of the study, and who has been diagnosed with TB. In index cases older than 7 years, only those with laboratory confirmed pulmonary TB were eligible; if 7 years or younger, we will include patients with TB diagnosed by a doctor without bacteriological confirmation, or bacteriologically-confirmed TB of any organ. Index cases will be diagnosed at any health facility in the study districts in the 6 weeks prior to recruitment. Severely ill and deceased patients will be included as index cases if we obtain written informed consent from a close family member. Severely ill patients with TB will be requested to confirm their participation in the study once they are capable of providing informed consent. We will exclude individuals who are incarcerated or are in long term in-patient care for reasons apart from TB.

Research teams will identify potentially eligible index TB cases by visiting hospital wards and clinics, from routine reporting of TB cases in public-sector health facilities in the two study districts (TB registers and notifications), and from lists of specimens positive for TB generated by public sector laboratories.

For each index TB case who provides informed consent, a baseline questionnaire will be completed; in paediatric index cases or where the index case is deceased or too ill to provide informed consent, this information will be obtained from the caregiver or participant’s representative. Additionally, we will ask index cases – or their representative – to complete a line-list of all household members.

### Design of interventions

The households of index TB cases will be randomly allocated in a 1:1 ratio to one of two interventions: **intensified household contact tracing and treatment support** (intervention); or **enhanced standard of care** (control).

### Randomisation, allocation and blinding

The unit of randomization is households of recruited index TB cases. The dwelling within which the household members live is defined as all rooms under a contiguous roofed area linked by doorways or windows through which air can pass. For this study, household members are defined as all individuals who shared dwelling airspace by either having slept overnight at least once, or shared at least two meals in the same household as the index case in the 14 days prior to the index case’s diagnosis of TB.

Index cases and their household members will be randomly allocated using a computer-generated allocation sequence stratified by district and with a block size of 10 generated prior to the first allocation by the study statistician. For each index case at both sites, allocation is accessed through a central study telephone line, staffed by a data manager in Johannesburg, unlinked to other aspects of the study. Randomisation only occurs after consent procedures and baseline questionnaires and after all eligibility criteria have been satisfied.

This is an open study as we cannot blind index cases, their households, or study field research teams to allocation. However, study research assistants are blinded to the allocation of households when they conduct outcome assessments, and investigator blinding will be maintained until final analysis; no interim analyses which reveal results of the study or provide an indication by group laboratory results, or other outcomes, will be provided to site staff or investigators.

### Intensified household contact tracing and treatment support (intervention group)

In households allocated to the intervention group, within 14 days of recruiting the index TB case, a research team comprising of an enrolled nurse and counsellors will conduct an initial household visit, record characteristics of the dwelling, seek consent to participate from all household members, and administer a short questionnaire to each recruited household member that will include: demographic details including relationship and exposure to the recruited index case; risk factors for TB; presence of TB symptoms; and history of previous and current HIV and TB treatment.

All household members will be offered confidential HIV testing and counselling in accordance with South African guidelines - or anonymised testing for study purposes if consent for this is provided - followed by blood sampling for CD4 cell count measurement in household members diagnosed HIV-infected. We will collect one spot sputum sample from each household member capable of producing a sputum sample (irrespective of the presence of TB symptoms), which will be transported to the public sector laboratory at each site, and tested using the GeneXpert MTB/Rif platform until approximately the end of 2017, when the Xpert Ultra was rolled out nationally in South Africa. Sputa will also be cultured using the liquid *Mycobacteria* growth indicator tube (MGIT) platform. Tuberculin skin testing (TST) will be offered to all household members without confirmed TB.

At a follow-up household visit 1 week later, results of sputum Xpert testing will be communicated in confidence to household members. Participants with microbiologically-confirmed tuberculosis or HIV requiring care and treatment will be supported to attend their nearest local primary care clinic to register for care and treatment. Household members will be offered isoniazid preventive therapy (IPT) according to South African guidelines - which have changed over the course of the trial (in brief those who test HIV-positive and whose sputum tests are negative for TB or those who are under 5 years of age). Additionally, we will offer IPT to HIV-seronegative individuals whose tuberculin skin test is positive (≥10 mm), and who agree to take isoniazid preventive treatment. Patients who accept isoniazid preventive treatment are dispensed the first month of isoniazid preventive therapy by study nurses at their home, and are referred for ongoing preventive treatment at their local clinics.

Because previous studies have demonstrated suboptimal rates of linkage into TB and HIV care [34, 35], a re-visit will be conducted 3 months after recruitment to support access to health facilities where required. If there has been no response to accessing care by individuals in a household, the local clinic will be approached to visit the household and take over their care.

### Enhanced standard of care (control group)

Each recruited index case – or their representative – allocated to the enhanced standard of care group will be provided with a referral letter and information materials for every household member they identify at baseline interview, and will be requested to give a pre-printed referral letter to each person in their household. The referral letter contains information about TB and HIV, recommends screening for TB and HIV, and provides details of local health facilities where screening and further care (if required) may be accessed. Additionally, the referral letter contains directions for health providers, including recommendations that TB screening, HIV testing and further care and prevention services (including ART and TB preventive therapy as required) should be offered in accordance with South African National guidelines because the individual was exposed to a likely infectious case of TB.

A week after recruitment, study research assistants will attempt to make a follow up telephone call to the index case, or their caregiver, to ensure the referral letters were given to household members, and to confirm that any ill household members have accessed their local health facility.

### Outcome measures

The primary outcome of the trial is a composite one: TB-free survival in the household starting 1 month after randomisation to the final ascertainment visit (an expected total follow-up time of ~ 14 months); the one-month lead-in time is to allow evaluation of the preventive effect of the study interventions, by excluding from analysis incident TB diagnoses that occur between months 0 and 1 that would likely detected as part of the intensified investigations in the intervention households. The follow-up time for those reaching the endpoint will be the time from a month after randomisation, or date of becoming a permanent household member (if entered the household at least 1 month after randomisation) to date of loss to follow-up or the date of the 15-month final outcome ascertainment visit; the follow-up time for those not reaching the endpoint will be the time from 1 month after randomisation or date of becoming a permanent household member (if entered the household at least 1 month after randomisation) to date of tuberculosis diagnosis or to date of death (or if both occur during the follow-up, the earliest of these). All analyses will be done using two different definitions of TB: (1) including all cases where TB was diagnosed or TB treatment started, irrespective of the diagnostic method (“all TB”); (2) including only those where hard copy of a laboratory confirmation of TB was seen by the study team or for whom there is written medical record confirmation of a positive laboratory test for TB (“microbiologically-confirmed TB”).

Secondary outcomes, together with their definitions, are:

1. Prevalence of TB infection at month 15 visit among household children who are aged under 14 years of age at the month 15 visit: Measured by tuberculin skin test (TST) reactivity – defined in three ways:
  - **TST diameter ≥ 10 mm** (irrespective of HIV serostatus or other comorbid condition) at the month 15 visit. This is our main definition of interest for this key secondary outcome.
  - **TST diameter ≥ 15 mm** in all participants irrespective of HIV serostatus, or other comorbid conditions at the month 15 visit. This is likely to be more specific.
  - **Differential TST diameter**: ≥5 mm in HIV infected children and in children who are underweight (defined as either height for age or weight for age Z-score of <− 2); TST diameter ≥ 10 mm in seronegative and non- malnourished children.

We will also report the distribution of TST readings in children < 14 years, and the estimated annual risk of TB infection (ARI).

2) Time between first onset of symptoms and initiation of anti-tuberculosis treatment, among household members diagnosed with TB between baseline and month 15 visit
3) HIV prevalence (reported as a percentage of those HIV-tested at the month 15 visit) of previously undiagnosed, or previously diagnosed, but untreated HIV infection
4) Cumulative incidence of all-cause mortality at the month 15 visit
5) HIV viral load, defined as the prevalence of detectable viraemia (> 400 copies per/ml) within each trial arm (should we have sufficient data from HIV-infected individuals to do this)
6) Estimation of the cost-effectiveness of the intensified household contact tracing and treatment support intervention compared to the enhanced standard of care from a societal perspective. Analyses for this outcome will be described in a separate economic statistical analysis plan.

#### Subgroup analyses

1. Subgroup analysis by study site (Capricorn District and Botshabelo District) will be conducted for all outcomes, in order to assess whether the effect of the intervention differs between a high (Botshabelo) and lower (Capricorn) TB burden setting.
2. For the secondary outcome of TB infection as assessed by TST reactivity among children aged < 14 years, we will conduct a subgroup analysis comparing children < 5 years of age with those aged ≥5 years at the month 15 visit.

### Outcome assessment

We will conduct an outcome household visit to all households, irrespective of their study allocation, at 15 months after the recruitment of the index case. Two months prior to the visit, we will attempt to locate the standard of care households (that have not been visited prior to this time) using a What3Words a mapping application – this to reduce the potential bias of travelling directly to the household versus attempting to find households for the first time at the outcome visit. Moreover, to reduce potential bias, study teams that have not visited that intervention household previously will be used to conduct the outcome visit.

At the outcome visit, Research Assistants blinded to the original allocation of the household will 1) trace all household participants, ascertain vital status and attempt to assess whether those who died, died with TB and/or HIV; 2) record episodes of TB and first HIV diagnosis, treatment and other hospitalizations from verbal report and inspection of patient-held records; 3) investigate participants with symptoms of TB (any of: cough, fever, weight loss, night sweats) by collecting sputum for smear microscopy, sputum culture for *M. tuberculosis* and Xpert; 4) offer repeat HIV testing to participants negative or not previously tested at baseline; and 5) conduct a prevalence survey for latent TB infection by testing all children under 14 years old with the tuberculin skin test.

### Health economics and cost-effectiveness

All costing and economic evaluations will be primarily from the perspective of the health care system to enhance comparability with other studies. However, estimates of household costs of illness and deaths will also be included in certain cost effectiveness measures to reflect a societal perspective. The analysis will also explore the magnitude of household costs, potential for catastrophic costs of HIV and TB, and implications for universal access.

We will use a direct costing approach to assess incremental costs of the intervention in achieving the primary objective. This will consider costs of diagnostic investigations, staff and travel and any other substantial items that may be identified. Intervention costs will be differentiated carefully from various services and also from research activities.

Interviews with household members will record direct and indirect costs (e.g. lost income) of illness and accessing healthcare, and about effect on various household asset holdings to explore dissaving and potential for catastrophic costs.

The principal cost effectiveness indicators will be incremental costs per person diagnosed with HIV and/or TB, incremental cost per person linked to HIV and/or TB care through the intervention in each setting, and incremental cost per incident TB case and death avoided. Further indicators such as estimated incremental cost per life-year saved or per TB treatment completion will be generated if the frequency and profile of reported clinical outcomes support this. In addition, we will measure health-related quality of life of household members at baseline and follow-up using the EuroQol EQ-5D [36].

Records of health service utilization (in-patient days and outpatient visits) for each group will be combined with unit costs for each type of care derived from step-down costings in relevant facilities to estimate possible cost savings and net costs.

Incremental cost effectiveness ratios will be compared to benchmarks for South African HIV, TB and other health interventions to facilitate interpretation for policymakers.

Sensitivity analyses will assess the strength and robustness of cost effectiveness findings, and aggregated and disaggregated unit cost measures will be reported. To clarify comparisons with other interventions and relevance for other settings, reports will clearly describe the case finding initiative and context, and explore system strengthening issues such as capacity needs, effects of scale and overall budget requirements.

### Statistical analysis plan

A sample size of 1200 index cases in each district (2400 in total) is proposed, based on previous studies in the districts, and assuming mean household size of 5.5 members, and household TB incidence that is the mean of that reported for each district. This will provide 80% power to detect a 30% difference (two-sided *p* < 0.05) in TB-free survival between the trial arms with an intra-cluster correlation coefficient of k = 0.3. Routine data from both sites suggest that they diagnose and treat at least double this number of TB cases per annum. No interim analysis will be undertaken.

Data will be captured using paper case record forms, and doubled entered into a secure RedCap Database hosted on a server at the University of Witwatersrand, Johannesburg, South Africa. To maintain confidentiality of participants, completed case record forms will be stored securely at study site research offices, and participant identifying information will be removed during data capture. Logical rules and check ranges for values will be implemented, and data quality checks will be run regularly by the study Data Manager.

All analysis will be done on an intention to treat basis, with denominators comprising households and household members randomly allocated to trial groups. We will compare baseline household-, and index case-, and household members-level socio-demographic and clinical characteristics between allocated trial groups. This will use absolute numbers and proportions for categorical variables. Means and standard deviations, or medians and interquartile ranges, will be used for continuous variables.

Outcomes will be assessed in two analysis populations: A) all household members who were listed at baseline for whom information is available at final study visit (“baseline cohort”, with analysis including and excluding TB index case conducted); B) all household members for whom information is available at final study visit irrespective of presence at baseline (“final visit cohort”). Cohort A will be the primary analysis population. Results from Cohort B will be used to investigate whether the effect of the intervention extends beyond those exposed to the intervention.

All analyses will be done on an intention-to-treat basis, i.e. data from household members included in populations A and B described above, will be included in the analysis, regardless of whether they underwent all intervention procedures in their trial arm.

Additionally, for secondary outcome 1, the analysis population will be restricted to those individuals aged less than 14 years at the time of outcome assessment at 15 months and for secondary outcome 2, the analysis population will be restricted to those individuals with a bacteriologically-confirmed TB diagnosis during the follow-up period.

For the primary trial outcome (TB free survival), Poisson regression with random effects to account for clustering at the household level, will be used to estimate a rate ratio, 95% confidence interval and *p*-value for the effect of the intervention. To allow for the stratified randomisation by district, a fixed effect term for district will be included in the regression model.

Analysis of secondary and exploratory outcomes will follow a similar approach, except where binomial outcomes are assessed (e.g. proportion of child household members with *MTB* infection; proportion of household members with undiagnosed or untreated HIV), in which case we will use logistic regression with random effects to account for household clustering. For secondary outcome 5 (community HIV load), we will calculate this as the fraction of HIV-positive individuals with a measured viral load who have detectable viraemia multiplied by the number of individuals testing HIV-positive and divided by the total population with a measured HIV status.

Subgroup analysis will be done by examining stratum-specific (district-specific) rate ratios (or odds ratios for binary outcomes) and by fitting terms for the interaction between trial arm and district in random effects regression models.

This trial offers a number of important health benefits, including early diagnosis and supported access to TB and HIV care, which are known to be problematic and expensive for individuals under current health systems in South Africa. Moreover, the trial will provide important information to assist in the development of improved HIV and TB services and pathways to care in local communities and in South Africa.

All index TB cases (or their representative if deceased) and household members will provide informed consent to participate in the study. Parental consent to participate in research will be obtained for children < 18 years and assent will be obtained from children 7-14 years. Individuals who are illiterate, or unable to write, will be invited to provide a thumbprint confirmation of consent to participant, accompanied by a witness signature.

Household tracing has the potential to inadvertently disclose confidential clinical information such as TB or HIV status, and to perpetrate stigma and discrimination, although previous cluster randomised trials in sub-Saharan Africa have demonstrated that these potential risks are minimal, with trial withdrawal due to stigma or discrimination extremely uncommon [37, 38]. Partner disclosure of HIV status and TB status have been clearly shown to be beneficial to household members and partners. Nevertheless, the trial team will take extensive steps to ensure that recruited index cases are fully aware of the possible implications of household tracing and follow-up, and they will be supported to disclose results to members of their household by trained research assistants and study nurses. HIV and TB testing will use South African National guideline procedures, which have been well-validated in terms of diagnostic accuracy.

## Discussion

The TB and HIV co-epidemics in sub-Saharan Africa continue to pose serious threats to public health, health systems and development. The End-TB Strategy has set out bold targets to identify and treat all individuals with TB to rapidly reduce incidence, prevalence, morbidity, and mortality, and eliminate the catastrophic costs associated with TB diagnosis and treatment [5]. Concerted action is underway to implement high impact HIV care and prevention to end the acquired immunodeficiency syndrome (AIDS) epidemic. Despite primary data [16, 17, 28], systematic reviews [12, 21] and guidelines [10] highlighting the high potential of index-case based case finding interventions for TB and HIV, implementation in the region has been limited by a lack of clear evidence on how cost-effective interventions are likely to be for households, communities and health systems. The present cluster-randomised trial will attempt to answer a series of critical outstanding questions for policymakers: can a strategy of household index TB case contact tracing, intensified screening for HIV and TB (with rapid molecular testing of all contacts), and supported linkage into HIV and TB treatment and prevention improve TB-free survival and reduce prevalence of *M. tuberculosis* infection in children?

Historically, household contact tracing of index TB cases – with an emphasis on prompt identification, screening and treatment of contacts of sputum-smear positive cases – has contributed to the substantial improvements in TB control over the twentieth century. This strategy is still widely used in high income settings [39]. However, concerns over resource-requirements and affordability have precluded implementation in low income settings. Moreover, where TB incidence and prevalence are high, and in high HIV prevalence settings, concerns have been raised that household-targeted case-finding approaches may be ineffective as incident TB disease among household contacts comprises only a small fraction of all population TB cases, meaning that household-targeted screening may only contribute marginally to case detection and overall reductions in disease incidence and prevalence [40]. Indeed, the ZAMSTAR trial did not clearly demonstrate the effectiveness of household-based interventions on reducing community TB prevalence and incidence of childhood infection [14]. However, coupled with emerging evidence of the higher than previously thought burden of previously undiagnosed HIV and TB among household contacts of TB cases [16], a number of new advances in more sensitive rapid molecular TB diagnostics (e.g. Xpert MTB/Rif), and increasing awareness of the effectiveness of longer duration TB chemoprophylaxis and ART have given fresh impetus to evaluate the effectiveness of more intensified household approaches.

Considerable uncertainty exists about where TB is predominantly transmitted in high HIV prevalence settings. Social mixing and genomic studies suggest that only a moderate fraction of transmissions occur within households, with settings outside the house such as churches, bars and public transport more commonly implicated [41–44]. Nevertheless, there remains a compelling public health and epidemiological case for targeting contact tracing towards the household contacts of index TB cases. Despite incident cases being infrequently linked by existing techniques at the population level, cohort studies have demonstrated that the risk of incident and prevalent TB is substantially and significantly higher among household contacts than among individuals not exposed to the household [29]. Moreover, household contacts are usually substantially easier to trace, screen and support to access care, treatment and prevention services than contacts encountered in other settings. Assessment of shared airspace exposure-time to potentially infectious cases can be more readily made, informing a more rational approach to screening criteria. Finally, the extremely high prevalence and incidence of previously undiagnosed and untreated HIV with high susceptible to TB disease makes an integrated household TB and HIV case-finding, screening, treatment and prevention approach feasible and attractive.

The units of randomisation in this trial are the households of index TB cases. In addition to facilitating delivery of trial interventions, the cluster-randomised design will allow us to determine effectiveness on TB-free survival at the household level, allowing us to test hypotheses around the impact on household-contacts. Should the present trial demonstrate intervention effectiveness, future trials may wish to investigate the effectiveness of interventions on TB incidence, prevalence or mortality at a community level. Such a cluster-randomised trial would however be very expensive, requiring large numbers of participants and clusters to evaluate the comparatively rare TB endpoints. Therefore, the present trial will provide important preliminary evidence on effectiveness to inform the design of future trials.

Strengths of this trial include the efficient design to evaluate the effectiveness on a number of important endpoints; the high likelihood that results will be directly informative to policymakers; and the potential to support capacity development at health facilities and university departments in traditionally under-resourced settings. There are however a number of challenges in the design and implementation. Despite recent advances, bacteriological TB diagnostics for active disease remain suboptimal, and screening for latent disease is limited by poor sensitivity, especially where environmental mycobacterial exposure and HIV are common. Because incident TB cases in household contacts may be bacteriologically-undetected, we will evaluate the effectiveness of interventions on incidence rates of both bacteriologically-confirmed and all-TB. Because of the nature of trial interventions, it is impossible to blind participants to allocation group. To minimise the risk of bias, research assistants conducting final outcome assessments will be blinded to household allocation, and investigator blinding will be maintained until the completion of all study activities and final data preparation. We have selected one very-high and one moderate TB incidence community for study sites, however analysis will be underpowered to detect differences in the impact of the intervention on outcomes between sites. Results will be generalizable to similar communities in sub-Saharan Africa, but may not be to other lower HIV prevalence settings.

In summary, results of this trial will provide policymakers with clear evidence about whether intensified household contact tracing of index TB cases, screening for HIV and TB, and supported linkage to care, treatment and prevention are effectiveness at improving TB free survival and reducing childhood *M. tuberculosis* prevalence, and can contribute to efforts to achieve the End-TB strategy goals.

## Supplementary information

**Additional file 1.** Map of study districts.

## Data Availability

The Principal Investigators (NAM, PM) and Statistician (ELW) will have access to the final trial dataset. Following unblinding and final analysis, study results will be formally presented to the District and Provincial health departments. Study results will be submitted for publication in a peer-reviewed journal with an emphasis of public health and tuberculosis soon after completion of analysis. Results will also be presented at an international scientific conference to facilitate dissemination. An anonymised final data set and code to allow reproducible analysis will be made publicly-available.

## Abbreviations

AIDS: Acquired immunodeficiency syndrome
ART: Antiretroviral therapy
CD4: Cluster of differentiation 4
DSMB: Data and safety monitoring board
HIV: Human immunodeficiency virus
IPT: Isoniazid preventive therapy
MGIT: *Mycobacteria* growth indicator tube
TB: Tuberculosis
TST: Tuberculin skin test
WHO: World Health Organization
ZAMSTAR: Zambia, South Africa Tuberculosis and AIDS Reduction
