# Supplementary figures and images for: Intensified household contact tracing, prevention and treatment support versus enhanced standard of care for contacts of tuberculosis cases in South Africa: study protocol for a household cluster-randomised trial
Source: BMC Infect Dis. 2019 Oct 12;19:839. doi: 10.1186/s12879-019-4502-5 (PMC6790042; doi:10.1186/s12879-019-4502-5)

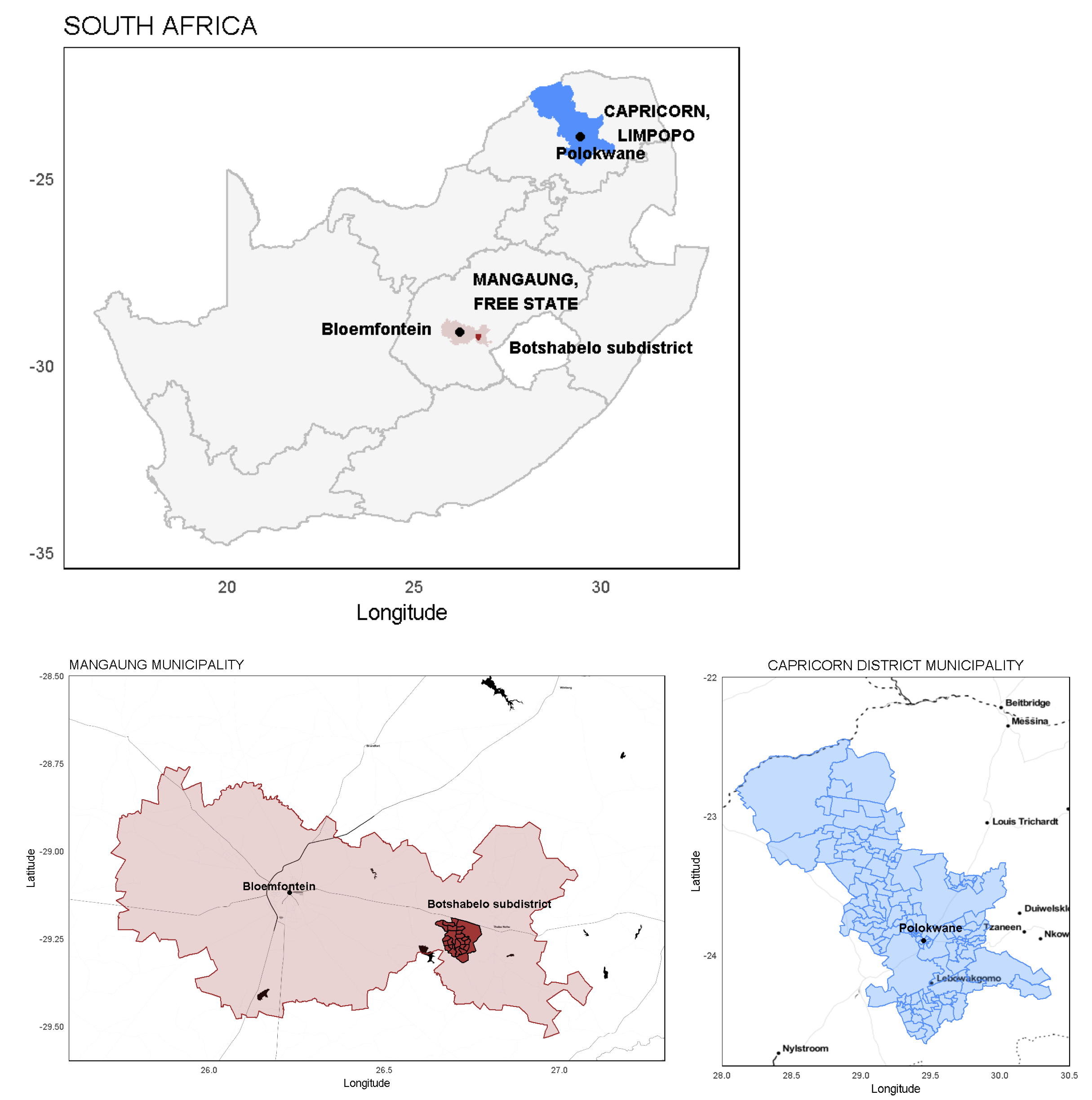

Supplement: Supplementary file 1 — Additional file 1. Map of study districts. [file 12879_2019_4502_MOESM1_ESM.tiff]
